# Incidence of postpartum and neonatal illnesses and utilization of healthcare services in rural communities in southern Ethiopia: A prospective cohort study

**DOI:** 10.1371/journal.pone.0237852

**Published:** 2020-08-27

**Authors:** Moges Tadesse Borde, Eskindir Loha, Bernt Lindtjørn

**Affiliations:** 1 School of Public Health, College of Medicine and Health Sciences, Hawassa University, Hawassa, Ethiopia; 2 Centre for International Health, University of Bergen, Bergen, Norway; 3 School of Public Health, College of Medicine and Health Sciences, Dilla University, Dilla, Ethiopia; 4 University of Bergen, Bergen, Norway; 5 Chr. Michelsen Institute, Bergen, Norway; Bradford Institute for Health Research, UNITED KINGDOM

## Abstract

Although improving postpartum and neonatal health is a key element of the Ethiopian health extension program, the burdens of postpartum and neonatal illnesses and healthcare-seeking in rural communities in Ethiopia are poorly characterized. Therefore, we aimed to assess the incidence and risk factors for these illnesses and measure the utilization of healthcare services. We conducted a prospective cohort study of 784 postpartum women and their 772 neonates in three randomly selected *kebeles* in rural southern Ethiopia. Eight home follow-up visits were conducted during the first 42 postpartum days, and six neonate follow-ups were conducted at the same home over the first 28 days of life. The Prentice, Williams, and Peterson’s total time Cox-type survival model was used for analysis. We recorded 31 episodes of postpartum illness per 100 women-weeks (95% confidence interval [CI]: 30%, 32%) and 48 episodes of neonatal illness per 100 neonate-weeks (95% CI: 46%, 50%). Anemia occurred in 19% of women (95% CI: 17%, 22%) and low birth weight (<2,500g) in 15% of neonates (95% CI: 13%, 18%). However, only 5% of postpartum women (95% CI: 4%, 7%) and 4% of neonate (95% CI: 3%, 5%) reported utilizing healthcare services. Walking over 60 minutes to access healthcare was a factor of both postpartum illnesses (AHR = 2.61; 95% CI: 1.98, 3.43) and neonatal illnesses (AHR = 2.66; 95% CI: 2.12, 3.35)). Birth weight ≥2500g was identified factor of neonatal illnesses (AHR = 0.39; 95% CI: 0.33, 0.46). Compared with younger mothers, older mothers with sick newborns (AHR = 1.22; 95% CI: 1.00, 1.50) or postpartum illnesses (AHR = 1.40; 95% CI: 1.03, 1.89) were more likely to seek healthcare. Reasons for not utilizing healthcare services included a belief that the illnesses were not serious or would resolve on their own, little confidence in the healthcare institutions, and the inability to afford the cost. The burden of postpartum and neonatal illnesses in rural communities of southern Ethiopia remains high. Unfortunately, few participants utilized healthcare services. We recommend strengthening the health system that enables identifying, managing, treating, and referring maternal and neonatal illnesses and provide reasonable healthcare at the community level.

## Introduction

Each year, approximately 60 million women worldwide experience pregnancy complications, and up to 20 million experience postpartum illnesses [1]. The postpartum period extends from one hour after delivery of the placenta to 42 days after childbirth (six weeks) [2]. Although 60% of maternal deaths happen during the postpartum period around the world [3], few studies focus on the occurrence and severity of postpartum illnesses in developing countries [4,5]. A small body of emerging literature suggests a high incidence of postpartum illnesses in these countries. For example, in India, about half of all pregnant women experience a postpartum illness [6], and more than 20% experience two or more postpartum illnesses [7]. In Pakistan, about 40% of women experience at least one type of postpartum illness [4]. The most common symptoms of postpartum illnesses are high fever (21%), heavy vaginal bleeding (14%), foul-smelling vaginal discharge (10%) [8], and breast problems (18%) [6]. The prevalence of postpartum anemia ranges from 50% to 80% [9]. Current data on the extent of postpartum hypertension are limited. However, in 2018, a review from Ethiopia indicated that the prevalence of hypertensive disorders during pregnancy is high (6%) [10]. Multiple socioeconomic and demographic factors, such as the cost of healthcare services and poverty, are linked with increased risk of postpartum illnesses [11].

Globally, 2.5 million neonates died in 2017 alone [12]. The neonatal period extends from birth to 28 days after birth (four weeks) [13]. Some studies indicate that one out of every 20 neonates worldwide dies of neonatal illnesses [14]. Most of these illnesses occur immediately after birth. In 2018, a study from Bangladesh indicated that 31% of neonates developed illness, and among those, 67% suffered from fever, 28% from difficult or fast breathing, 28% from low temperature, 13% from poor sucking or feeding, 10% had chest in-drawing, and 37% had more than one of these symptoms [15]. In 2019, 31% of mothers reported neonatal jaundice or low birth weight in their children [16]. Low birth weight <2500g can result from preterm birth (i.e., gestation <37 completed weeks), intrauterine (fetal) growth restriction, or both, and is measured in the first 48 hours of life [17]. Potential factors associated with neonatal illnesses include geographic location; household wealth status; and the mother’s marital status, age, education, and occupation [8]. Mothers’ and caregivers’ perceptions about these illnesses and the cost of treatment can influence their healthcare-seeking behavior [11], which in turn affects neonates’ utilization of healthcare services.

In 2003, Ethiopia implemented a health extension program to improve access to and quality of healthcare in rural communities [18]. Yet, identifying and treating postpartum and neonatal illnesses and increasing the use of healthcare services in these populations remain key challenges of the program [19]. Approximately 70% of pregnant women still deliver at home, and healthcare-seeking for ill neonates has remained low. In 2009, illnesses were reported among 26% of neonates in Ethiopia [20]. By 2016, low birth weights still occurred in 13% of children, only 17% of women received care after childbirth, and about 70% of women with postpartum illnesses could not afford healthcare [21]. Therefore, this study aimed to assess the incidence and risk factors for postpartum and neonatal illnesses and measure the utilization of healthcare services in rural communities in southern Ethiopia. The findings might help policymakers address issues related to postpartum and neonatal illnesses and improve the low utilization of healthcare services.

## Materials and methods

A prospective cohort study of 784 women who had recently given birth and their 772 neonates was conducted from May 2017 to July 2018 in three randomly selected *kebeles* (i.e., *Mekonisa*, *Hase-Haro*, *and Tumata-Chiricha*) in the *Wonago* district of southern Ethiopia, which is located 420 km from the city of Addis Ababa. Each woman was followed up eight times at home during her first 42 postpartum days, and each neonate was followed up six times at the same home over the first 28 days of life. The details of this cohort are described in our previous study about pregnancy-related illnesses [22]. During our study, some women and neonates could not be contacted because of social unrest in a small part of our study area and were therefore excluded.

We defined postpartum illnesses as any disorder after childbirth that hurt a woman’s health [23]. Neonatal illnesses were similarly defined as any disturbance of the normal state of the body and organs of the neonate [16]. We defined utilization of healthcare services as any use of healthcare services or any visit to a healthcare facility to get outpatient and inpatient healthcare services to treat postpartum or neonatal illnesses [24]. We defined healthcare services according to the Encyclopedia of Behavioral Medicine as “an array of medical care or services that are recognized under state law and are performed by healthcare professionals or under their direction, for promoting, maintaining, or restoring health to those in need (i.e., patients, families, and communities) in either of all settings of care (i.e., health posts, health centers, hospitals, and homes)” [24].

In Ethiopia, the health service is restructured into a three-tier system: primary, secondary, and tertiary level of healthcare, and the healthcare system is organized based on the type of care provided [25]. In the primary tier system; the primary healthcare unit consists of a health center and five satellite health posts. One health center is for 15,000–25,000 people in rural areas, and 40,000 people in urban areas and each health post is for 3,000–5,000 people in rural areas. The primary hospital serves 60,000–100,000 people. The secondary tier system includes general hospital which is for 1–1.5 million people, and the tertiary tier system also includes a specialized hospital that is for 3.5–5 million people.

The primary tier system embraces all the healthcare services provided at all levels, and at health posts, most primary healthcare services are provided by health extension workers. These lay individuals are not nurses or trained healthcare professionals, although they have received one-year training in primary healthcare. Health extension workers can address issues related to infectious diseases (e.g., tuberculosis, malaria), communicable and sexually transmitted infections (e.g., HIV/AIDS), maternal and child health, common nutritional disorders, hygiene, and environmental health, immunization and family planning, and reproductive health. Most pregnant women in Ethiopia who seek healthcare use their local health posts. If the case is serious, the Health Extension Worker may refer them to a health center [25].

In our study, women with postpartum illnesses were identified based on symptoms and clinical measurements, such as hemoglobin and blood pressure levels [23], recorded by trained data collectors. Hemoglobin was measured at the end of the 6-week postnatal period using the HemoCue analyzer Hb 301 System (www.hemocue.com). We defined anemia as a hemoglobin value of <12 g/dL at six weeks after childbirth [26]. Blood pressure was measured during each visit using a Riester ri-champion N digital apparatus (www.riester.de). High blood pressure was defined as >140/90 mm Hg [27].

Neonates with neonatal illnesses were identified based on reported symptoms by the mother and some measurements, such as neonates’ weight and length were measured using the Health O Meter® Portable Home Care Baby Scale (Pelstar, LLC; www.chichestershomecare.net). Neonatal mortality was defined as the death of the neonate within the first 28 days of life. Low birth weight was defined as <2,500g, measured within the first 48 hours of life. As birth weight is closely associated with neonatal mortality and morbidity and is used as a public health indicator [28], we recorded birth weight as an exposure variable together with other neonatal illnesses [28].

### Outcome variables

We assessed two primary outcomes for mothers and their neonates: illnesses and utilization of healthcare services. We measured these outcomes in two ways: as a count and as a dichotomous value (0 = no, 1 = yes). We assessed 10 symptoms and signs of postpartum illnesses [29,30]: hemorrhage, high fever, foul-smelling vaginal discharge, blurred vision with severe headache, severe abdominal pain, urinary incontinence, breast pain and engorgement, severe tiredness, anemia, and hypertension. S1 Table summarizes the symptoms of postpartum illnesses. We also assessed 12 symptoms and signs of neonatal illnesses [30,31]: not sucking properly, high fever (≥37.5°C), diarrhea, cord stump with redness or pus, persistent vomiting, hypothermia (≤35.5°C), fast breathing (≥60 breaths per minute), severe chest in-drawing, no spontaneous movement, jaundice, red or discharging eye, and lethargy. S2 Table summarizes the symptoms of neonatal illnesses.

### Exposure variables

The exposure variables for illnesses included participant characteristics and community-level variables. The women’s basic characteristics included her age, age at first marriage, age at first birth, birth interval (≥2 years or <2 years between births), occupation (domestic service or other), household wealth index, total monthly household expenditure (more or less than $30), gravidity, parity, history of abortion, history of stillbirth, marital status, and educational status. The neonates’ exposure characteristic was the birth weight (≥2500g or <2,500g). Low birth weight <2,500g was regarded as an exposure variable. Community-level variables included the type of road (asphalt or other) and walking distance to the nearest health facility. The exposure variables for the utilization of healthcare services were postpartum and neonatal illnesses and all previously listed exposure variables.

### Quantitative variables

Continuous variables were assessed for symmetry, and parametric tests were used for normally distributed variables.

### Sample size

To assess the sample, we made some assumptions based on our previous study [22]. We assumed a 15.5% incidence of illnesses among pregnant women and a 1.65 relative risk among poor women, compared with rich women (95% confidence level [CI], 80% power, and 1:1 ratio of unexposed to exposed) [32]. The sample size was estimated at 898 after adding 10% for non-responses. S1 Fig summarizes the flowchart of the recruitment of participants.

During the initial recruitment, 898 pregnant women were included in the study. Of those, 86 were excluded due to incomplete data, 14 had abortions, two were not pregnant, and instead had ovarian cysts, one died in an accident, and one refused to participate. Thus, 794 pregnant women were included in the analysis of pregnancy-related illnesses. During the follow-up visits, 10 women with incomplete data were excluded. The final sample thus included 784 postpartum women (S1 Fig).

Neonates born from 794 pregnant women were eligible for inclusion in the study. Out of 808 births, 782 were live births and 26 were stillbirths. Thirteen women had multiple births, and 781 women had singleton births. Of the 782 live births, we excluded 10 due to incomplete data, leaving a final sample of 772 neonates (S1 Fig).

### Data collection

Baseline socioeconomic and demographic data and follow-up data were collected via a questionnaire during visits to participants’ homes. Data from postpartum women were collected eight times: within 24 hours and within 24–72 hours after birth and at the end of the first, second, third, fourth, fifth, and sixth weeks. The questionnaire was adapted from the WHO Maternal Morbidity Measurement Tool [33]. Data from neonates were collected six times at the same home visits: within 24 hours and within 24–72 hours of birth and at the end of the 7^th^, 14^th^, 21^st^, and 28^th^ days. The questionnaire was adapted from the Johns Hopkins University Tools and Indicators for Maternal and Newborn Health [34].

Postpartum and neonatal questionnaires were prepared in English, translated into the local Gedeo (see S1 File Gedeo questionnaire for postpartum women and neonates.rar) and Amharic (see S2 File Amharic questionnaire for postpartum women.rar and S4 File Amharic questionnaire for neonates.rar) languages, and then translated back into the English language (see S3 File English questionnaire for postpartum women.rar and S5 File English questionnaire for neonates.rar).

The trained data collectors read the symptoms aloud and then asked the women to indicate whether they or their neonates had any of the symptoms, whether they utilized healthcare services, and reasons why they did not seek healthcare during illnesses. To assess the need for the utilization of healthcare services during postpartum and neonatal illnesses, a community-based approach was used rather than a facility-based approach [35].

### Statistical methods

The data were entered in EpiData version 3.1 software (EpiData Association, Odense, Denmark). Descriptive statistical analysis was used to determine the distribution of illness incidences and the utilization of healthcare services. In this paper, one statistical model was used to analyze recurrent event data. The interpretation of results and analyses of risk factors were based on Prentice, Williams, and Peterson’s total time Cox-type survival model [36,37]. This model is a robust option for recurrent events of illnesses and the utilization of healthcare services. To control for the effect of missing values, the analysis was restricted to women and neonates with complete data [38]. Correlations among variables during pregnancy and postpartum periods or during neonatal and pregnancy periods also were assessed. STATA software version 15 was used for analysis (Stata Corp LLC, College Station, TX, USA). Detailed information on the methods, study design, procedures, sample size, statistical methods, and major findings of illnesses during pregnancy are presented in our previous study [22].

### Ethical considerations

This study was approved by the institutional ethical review board at Hawassa University, College of Medicine and Health Sciences (IRB/100/08), and by the Regional Committees for Medical and Health Research Ethics of Western Norway (2016/1626/REK vest). Written permission was obtained from the *Gedeo* Zone health department and the *Wonago* district health office. Written informed consent was obtained from each woman after she received an explanation of the purpose of the study. The privacy, anonymity, and confidentiality of all participants were maintained. If a data collector observed any illness among participants during the study period, they tried to link the mother or child with health extension workers in the *kebele*.

## Results

### Background characteristics of study participants

A total of 794 postpartum women and their 782 neonates participated in our study. However, 10 women and 10 neonates had incomplete data and were excluded from the study, leaving 784 women and 772 neonates in the analysis. The response rate was 99% (784 of 794 postpartum women and 772 of 782 neonates), as shown in S1 Fig. From 794 pregnant women, 13 women had multiple births and 781 women had singleton births, and we registered 808 (782 live and 26 still) births. Among participating neonates, 751 were from singleton births, and 21 were from multiple births.

Table 1 presents the participants’ characteristics. Almost two-thirds of the women (509, 64.9%) delivered at home, and 96.3% (490 of 509) of those were attended by family members. Approximately 23.6% (185 of 784) reported that their most recent pregnancy was their first pregnancy. Vaginal deliveries accounted for 98.6% (773 of 784) of deliveries. About 13% (102 of 772) of neonates were born to mothers aged 15–19 years, and 57% (439 of 772) were born to mothers whose age at first birth was younger than 19 years. Nearly 63% (484 of 772) were born to mothers who did not have any formal education. The mean weight of neonates at birth was 2,890g (range: 2000g–4500g).

**Table 1:** Characteristics of study participants in rural southern Ethiopia, May 2017 to July 2018.

Socioeconomic characteristics		Postpartum women (n = 784)		Neonates (n = 772)	
		Frequency	Percent	Frequency	Percent
*Kebele*/residence	Mekonisa	381	48.6	374	48.4
	Hase-Haro	225	28.7	224	29.1
	Tumata-Chiricha	178	22.7	174	22.5
Women’s age in years	15–19	103	13.1	102	13.2
	20–24	223	28.4	222	28.8
	25–29	283	36.1	281	36.4
	30–34	134	17.1	125	16.2
	35+	41	5.2	42	5.4
Women’ marital status	Ever married	782	99.7	766	99.2
	Not married	2	0.3	6	0.8
Women’s educational status	Had formal education	284	36.2	288	37.3
	Had no formal education	500	63.8	484	62.7
Women’s occupation	Domestic service	725	92.5	702	90.9
	Others	59	7.5	70	9.1
Household wealth index	Rich	452	57.7	451	58.4
	Poor	332	42.3	321	41.6
Type of road to the nearest health facility	Asphalt	206	26.3	201	26.0
	Others	578	73.7	571	74.0
Walking distance to the nearest health post in minutes	<30	434	55.4	426	55.2
	30+	350	44.6	346	44.8
Walking distance to the nearest health center in minutes	<40	511	65.2	508	65.8
	40+	273	34.8	264	34.2
Walking distance to the nearest hospital in minutes	<60	399	50.9	397	51.4
	60+	385	49.1	375	48.6
Total household total expenditure per month	<$30	194	24.7	192	24.9
	$30+	590	75.3	580	75.1
Women’s age at first marriage in years	10–14	1	0.1	1	0.1
	15–19	705	89.9	694	89.9
	20–24	77	9.8	76	9.8
	25–29	1	0.1	1	0.1
Women’s age at first birth in years	15–19	447	57.0	439	56.9
	20–24	332	42.3	328	42.5
	25–29	5	0.6	5	0.6
Women’s gravidity (no of pregnancy)	Multigravida	604	77.0	594	76.9
	Primigravida	180	23.0	178	23.1
Women’s parity (no of birth)	Multipara	602	76.8	591	76.6
	Nullipara	182	23.2	181	23.4
Birth interval in years	<2	380	48.5	374	48.4
	2+	404	51.5	398	51.6
Mother’s history of abortion	Yes	47	6.0	47	6.1
	No	737	94.0	725	93.9
Mother’s history of stillbirth	Yes	36	4.6	36	4.7
	No	748	95.4	736	95.3
Birth type	Singleton	-	-	751	97.3
	Twin	-	-	18	2.3
	Triplet	-	-	3	0.4
Sex of neonates	Male	-	-	394	51.0
	Female	-	-	378	49.0
Weight of neonates at birth in grams	≥2500	-	-	654	84.7
	<2500	-	-	118	15.3
The length of neonates at birth in cm	≤43.0	-	-	445	57.6
	≥43.1	-	-	327	42.4

N.B. 1 USD = 27.64 ETB on August 31, 2018; Poor included the 1^st^, 2^nd^, and 3^rd^ quintiles, rich included the 4^th^ and 5^th^ quintiles in the wealth index.

### Incidence of postpartum illnesses

Table 2 presents the occurrence of postpartum illnesses, utilization of healthcare services, and women’s reasons for not utilizing healthcare services. In the six follow-up weeks, 914 illnesses episodes (minimum = 1, maximum = 25) were recorded. The incidence of illnesses was 31 per 100 postpartum women (95% CI: 30.0%, 32.3%) with 1,952 events or illnesses over 6,272 visits (i.e., total analysis time at risk). Among women, 31% (244 of 784) experienced at least one type of postpartum illness. The most common problems reported were anemia, blurred vision with headache, excessive vaginal bleeding, severe abdominal pain, and hypertension. Among all women in the analysis, 3.4% (27 of 784) had foul-smelling vaginal discharge (95% CI: 2.3, 4.9), 2.8% (22 of 784) had breast pain and engorgement (95% CI: 1.8, 4.2), and 0.6% (5 of 784) had high fever (95% CI: 0.2, 1.4). Also, 19% (148 of 784) were anemic (95% CI: 16.4, 21.9), but no iron or folic acid supplementation was administered during the study period. The incidence of hypertension was 3.4% (27 of 784) among the postpartum women (95% CI: 2.3, 4.9).

**Table 2:** Postpartum illnesses, utilization of healthcare services, and reasons for not utilizing healthcare services in rural southern Ethiopia, May 2017 to July 2018.

Postpartum illnesses	# women with ≥1 illness	%	# women without illnesses	# illnesses episodes	# events for the utilization of healthcare services (episodes)		# illnesses episodes per woman	The reasons cited for not utilizing healthcare service at least once										
								Did not perceive the presence of illnesses		Waited for spontaneous recovery		The illness was not serious		Lack of money		Lack of trust		Total
					Yes	%	No 95% CI*	Yes	%	Yes	%	Yes	%	Yes	%	Yes	%	
Anemia	149	19.0	635	149	1	0.7	1.0	2978	83.1	538	61.2	1467	63.8	35	17.3	5	17.2	5023
Blurred vision with headache	70	16.1	714	299	15	5.0	4.3 (3.8,4.8)	180	5.0	113	12.9	169	7.4	43	21.3	5	17.2	505
Excessive vaginal bleeding	62	14.2	722	159	2	1.3	2.6 (2.2,3.0)	104	2.9	98	11.1	217	9.4	41	20.3	6	20.7	466
Severe abdominal pain	38	8.7	746	96	12	12.5	2.5 (2.1,2.9)	122	3.4	26	3.0	106	4.6	24	11.9	5	17.2	283
Foul-smelling vaginal discharge	27	6.2	757	51	2	3.9	1.9 (1.6.2.3)	46	1.3	28	3.2	95	4.1	27	13.4	5	17.2	106
Urinary incontinence	27	6.2	757	47	2	4.3	1.7 (1.4,2.0)	38	1.1	47	5.3	88	3.8	20	9.9	3	10.3	196
Hypertension (≥140/90 mm Hg)	27	6.2	757	34	0	0.0	1.3 (0.9,1.5)	0	0.0	6	0.7	23	1.0	2	1.0	0	0.0	31
Breast pain and engorgement	22	5.0	762	48	3	6.3	2.1 (1.8,2.6)	56	1.6	11	1.3	102	4.4	7	3.5	0	0.0	176
Fatigue or tiredness	9	2.1	775	25	9	36.0	2.8 (2.4,3.2)	41	1.1	7	0.8	17	0.7	2	1.0	0	0.0	67
High fever	5	1.1	779	6	0	0.0	1.2 (0.9,1.5)	20	0.6	5	0.6	14	0.6	1	0.5	0	0.0	40
Total	436^1^	31.1^2^		914^3^	46^4^	5.0^5^	2.1 (1.8,2.6)^6^	3585^7^	51.3	879^7^	12.6	2298^7^	32.9	202^7^	2.9^7^	29^7^	0.4	6993^8^

*Number of episodes of illnesses divided by the number of women with illnesses per row

^1^Total number of women with illnesses

^2^Total number of women with illnesses divided by the total number of women in the study

^3^Total episodes of illnesses

^4^Total episodes of the utilization of healthcare service

^5^Total episodes of the utilization of healthcare service (46) divided by total episodes of illnesses (914)

^6^Total episodes of illnesses divided by the total number of women with illnesses

^7^Total number of reasons in each column divided by the overall number of reasons (e.g., 3585/6993 for waited for spontaneous recovery).

### Utilization of healthcare services during postpartum illnesses

We recorded 46 episodes of use of healthcare services, or 6.4 uses per 1,000 postpartum women (95% CI: 4.6, 8.6), and 40 events of the utilization of healthcare services over 6,272 visits (i.e., total analysis time at risk). Only 2.0% (5 of 244) of the women who had symptoms of illnesses utilized healthcare services at least once during their illnesses.

The rate of utilization of healthcare service for any episode of illnesses was only 5% (95% CI: 3.8%, 6.6%), or 46 out of 914 illnesses episodes. For symptoms of infection, healthcare services were utilized for foul-smelling vaginal discharge in only 3.9% (2 of 51) episodes. Furthermore, only 6.3% (3 of 48) postpartum women with breast pain and engorgement utilized healthcare services. The main reasons reported by the women for not using healthcare services were that they did not perceive the illnesses as a problem (51.3%; 3,585 of 6,993 responses), they thought the illnesses would resolve on its own (12.6%; 879 of 6,993 responses), they thought the illnesses were not serious (32.9%; 2,298 of 6,993 responses), they could not afford to visit the healthcare institutions (2.9%; 202 of 6,993 responses), and they lacked confidence in the healthcare facilities (0.4%; 29 of 6,993 responses).

The rate of utilization of healthcare services for anemia was only 0.7% (1 of 149 episodes). The main reasons for not utilizing healthcare services for anemia were that the women did not perceive anemia as a problem (59.3%; 2,978 of 5,023 responses), they thought anemia would resolve on its own (10.7%; 538 of 5,023 responses), they thought anemia was not a serious health problem (29.2%; 1,467 of 5,023 responses), they could not afford to visit the healthcare institutions (0.7%; 35 of 5,023 responses), or they lacked confidence in the healthcare facilities (0.1%; 5 of 5,023 responses). Although 2.4% of women after childbirth were hypertensive, this problem was not associated with any utilization of healthcare services (Table 2).

### Determinants of postpartum illnesses

Table 3 presents the results of Prentice, Williams, and Peterson’s total time Cox-type survival model analysis of postpartum illnesses. Compared to the other women in this study, those who had to walk more than 60 minutes to access the nearest hospital were three times more likely to experience an illness episode during the reporting period (adjusted hazard ratio [AHR] = 2.61; 95% CI: 1.98, 3.43).

**Table 3:** Prentice, Williams, and Peterson’s total time Cox-type survival model analysis for postpartum illnesses in rural southern Ethiopia, May 2017 to July 2018.

	Status of total events (postpartum illnesses as Yes/No) (n = 6,272 women-weeks)		Crude hazard ratio (95% CI)	p-value	Adjusted hazard ratio (95% CI)	p-value
	Yes	No				
Women’s age (years)	1952	4320	1.00 (0.97, 1.02)	0.731	-	-
Women’s age at first marriage (years)	1952	4320	1.05 (0.97, 1.34)	0.203	-	-
Women’s age at first birth (years)	1952	4320	1.04 (0.97, 1.12)	0.274	-	-
Birth interval (years)						
2+	984	2248	0.96 (0.78, 1.78)	0.673	-	-
<2	968	2072	1.0		-	-
Women’s occupation						
Other (daily laborer, farming, etc)	104	368	0.69 (0.42, 1.13)	0.142	-	-
Domestic service	1848	3952	1.0		-	-
Household wealth index						
Rich	1128	2488	1.01 (0.81, 1.24)	0.959	-	-
Poor	824	1832	1.0		-	-
Total household monthly expenditure						
$30+	1930	4208	1.41 (0.59, 3.37)	0.440	-	-
<$30	32	112	1.0		-	-
Gravidity						
Multigravida	1480	3352	0.93 (0.73, 1.19)	0.582	-	-
Primigravida	472	968	1.0		-	-
Parity						
Multipara	1464	3352	0.91 (0.72, 1.50)	0.421	-	-
Nullipara	488	968	1.0		-	-
History of abortion						
Yes	96	280	0.81 (0.49, 1.34)	0.412	-	-
No	1856	4040	1.0		-	-
History of stillbirth						
Yes	120	168	1.36 (0.91, 2.03)	0.132	-	-
No	1832	4152	1.0		-	-
Type of road to the nearest health facility						
Asphalt	464	1184	0.88 (0.68, 1.12)	0.292	-	-
Other	1488	3136	1.0		-	-
Walking distance to the nearest health post in minutes						
30+	1008	1792	1.32 (1.08, 1.63)	0.008	-	-
<30	944	2528	1.0		-	-
Walking distance to the nearest health center in minutes						
40+	752	1432	1.17 (0.95, 1.45)	0.140	-	-
<40	1200	2888	1.0		-	-
Walking distance to the nearest hospital in minutes						
60+	1264	1816	1.90 (1.52, 2.38)	0.001	2.61 (1.98, 3.43)	0.001
<60	688	2504	1.0		1.0	

Significant at p<0.05; CI = confidence interval.

### Incidence of neonatal illnesses

Table 4 presents the occurrence of neonatal illnesses, utilization of healthcare services, and the mother’s reason for not utilizing healthcare services. In the 28 follow-up days, 1,624 neonatal illness episodes (minimum = 1, maximum = 19) were recorded. The incidence of illnesses was 48 per 100 neonate weeks (95% CI: 45.9%, 49.8%), with 2,214 events or illnesses over 4,632 visits (i.e., total analysis time at risk). There were 14 neonatal deaths (i.e., 19 per 1,000 live births) due to severe chest in-drawing, high fever, low birth weight, fast breathing, or umbilical cord infection, and unknown reasons (accidental).

**Table 4:** Neonatal illnesses, utilization of healthcare services, and reasons for not utilizing healthcare services in rural southern Ethiopia, May 2017 to July 2018.

Neonatal illnesses	# neonates with > = 1 illnesses				# neonates without illnesses	# illnesses episodes	# events for utilization of healthcare services (episodes)		# illnesses episodes per neonate	The reasons cited for not utilizing the healthcare service at least once								
										Neonate did not have an illness		Waited for spontaneous recovery		The illness was not serious		Lack of money		Total
	Alive		Died				Yes	%	No (95% CI)*	Yes	%	Yes	%	Yes	%	Yes	%	
	Yes	%	N	%														
Not sucking well	272	35.2	0	0	500	1,412	8	0.6	5.2 (4.8,5.6)	119	39.1	6	33.3	17	17.9	1	5.0	143
High fever	8	1.0	3	0.4	761	15	6	40.0	1.4 (1.1,1.7)	15	4.9	0	0.0	0	0.0	2	10.0	17
Diarrhea	10	1.3	0	0	762	11	11	100.0	1.1 (0.9,1.3)	33	10.9	0	0.0	6	6.3	3	15.0	42
Red cord stump or with pus	7	0.9	2	0.3	763	12	2	16.7	1.3 (1.1,1.6)	12	3.9	0	0.0	6	6.3	2	10.0	20
Persistent vomiting	8	1.0	0	0	764	12	8	66.7	1.5 (1.3,1.7)	11	3.6	0	0.0	5	5.3	1	5.0	17
Low body temperature	8	1.0	0	0	764	15	6	40.0	1.9 (1.6,2.2)	16	5.3	0	0.0	5	5.3	3	15.0	24
Fast breathing	4	0.5	2	0.3	766	9	2	22.2	1.5 (1.2,1.8)	6	2.0	0	0.0	5	5.3	1	5.0	12
Severe chest in-drawings	2	0.3	3	0.4	767	5	3	60.0	1.0	10	3.3	2	11.1	6	6.3	1	5.0	19
No spontaneous movement	3	0.4	0	0	769	8	2	25.0	2.7 (2.4,3.0)	0	0.0	0	0.0	5	5.3	1	5.0	6
Yellowish eye, skin, sloe (jaundice)	3	0.4	0	0	769	3	3	100.0	1.0	4	1.3	0	0.0	5	5.3	3	15.0	12
Red or discharging eye	2	0.3	0	0	770	2	2	100.0	1.0	0	0.0	0	0.0	5	5.3	1	5.0	6
Lethargy	2	0.3	0	0	770	2	2	100.0	1.0	0	0.0	0	0.0	5	5.3	1	5.0	6
Unspecified	-	-	1	7.1	-	-	-	-	-	-	-	-	-	-	-	-	-	-
Total	329^1^	48^2^	11	1.4		1,506^3^	55^4^	3.7^5^	3.7(3.2,4.2)^6^	226^7^	69.6	8	2.5	70	21.6	20	6.2	324^8^

*Number of episodes of illnesses divided by the number of neonates with illnesses per row

^1^Total number of neonates with illnesses

^2^Total number of neonates with illnesses divided by all number of neonates in the study

^3^Total episodes of illnesses

^4^Total episodes of the utilization of healthcare services

^5^Total episodes of the utilization of healthcare services (56) divided by total episodes of illnesses (1,624)

^6^Total episodes of illnesses divided by the total number of neonates with illnesses

^7^Total number of reasons in each column divided by

^8^overall number of reasons (e.g., 304/437 for waited for spontaneous recovery).

During the follow-up, the most common problems reported were not sucking well, high fever, diarrhea, and cord stump with redness or pus. Among neonates with severe illnesses, 1.2% (9 of 772) had cord stump with redness or pus (95% CI: 0.6, 2.1), 0.8% (6 of 772) had fast breathing (95% CI: 0.3, 1.6), and 1.4% (11 of 772) had high fever (95% CI: 0.8, 2.5). In addition, 0.4% (3 of 772) had signs of jaundice (95% CI: 0.1, 1.1), and 1.3% (10 of 772) experienced diarrhea (95% CI: 0.7, 2.3). Among those with feeding problems, 35% (272 of 772) were not able to suck well (95% CI: 31.9, 38.7), and 1% (8 of 772) had persistent vomiting (95% CI: 0.5, 2.0).

### Birth weight of neonates

Among neonates, 15% (118 of 772) had low birth weights <2500g (95% CI: 12.9, 18.0). However, only 0.9% (1 of 118) with low birth weight utilized healthcare services. Many mothers did not perceive low birth weight as a problem (69%; 78 of 113 responses), or they thought that low birth weight would resolve on its own (9%; 10 of 113).

### Utilization of healthcare services during neonatal illnesses

We recorded 55 episodes of the utilization of healthcare services and 60 events of the utilization of healthcare services over 4,632 visits (i.e., total analysis time at risk). Only 2.4% (9 of 369) of ill neonates utilized healthcare services at least once during illnesses. The rate of utilization of healthcare services for any illness episode was only 3.7% (95% CI: 2.8%, 4.7%), or 55 out of 1,506 illnesses episodes. For signs of severe neonatal illnesses (i.e., cord stump with redness or pus, fast breathing, severe chest in-drawing, and high fever), only 17% utilized healthcare services for cord stump issues (2 of 12 episodes), 60% for severe chest in-drawing (3 of 5 episodes), and 22% for fast breathing (2 of 9 episodes). For signs of feeding problems, only 0.6% (8 of 1,412 episodes) utilized healthcare services. The main reasons for not utilizing healthcare services were that mothers did not perceive the illnesses as a problem (70%; 304 of 437) or could not afford to visit the healthcare institutions (4.6%; 20 of 437). Table 3 shows the results.

### Determinants of neonatal illnesses

The Prentice, Williams, and Peterson’s total time Cox-type survival model analysis showed that neonates weighing ≥2500g at birth were 0.39 times less likely to be ill (AHR = 0.39; 95% CI: 0.33, 0.46). Neonates born to mothers who walked more than 60 minutes to access the nearest hospital were three times more likely to experience an illness episode during the study period (AHR = 2.66; 95% CI: 2.12, 3.35). Table 5 shows the results.

**Table 5:** Prentice, Williams, and Peterson’s total time Cox-type survival model analysis for neonatal illnesses in rural southern Ethiopia, May 2017 to July 2018.

Exposure variables	Prentice-Williams-Peterson total time survival analysis					
	Status of total events (neonatal illnesses as Yes/No) n = 4,632 person-time		Crude hazard ratio (95% CI)	p-value	Adjusted hazard ratio (95% CI)	p-value
	Yes	No				
Mother’s age (years)	2,214	2,418	1.00 (0.98, 1.01)	0.861	-	-
Mother’s age at first marriage (years)	2,214	2,418	0.90 (0.84, 0.97)	0.007	-	-
Mother’s age at first birth (years)	2,214	2,418	0.97 (0.91, 1.03)	0.294	-	-
Birth interval (years)						
2+	1,152	1,236	1.02 (0.88, 1.18)	0.799	-	-
< 2	1,062	1,182	1.0		-	-
Mother’s occupation						
Other (daily laborer, farming, etc.)	234	186	1.19 (0.95, 1.48)	0.136	-	-
Domestic service	1,980	2,232	1.0		-	-
Household wealth index						
Rich	1,386	1,320	1.19 (1.02, 1.39)	0.027	-	-
Poor	828	1,098	1.0		-	-
Total household monthly expenditure						
$30+	1,620	1,860	0.90 (0.77, 1.06)	0.218	-	-
<$30	594	558	1.0		-	-
Mother’s gravidity						
Multigravida	1,704	1,860	1.00 (0.84, 1.19)	0.989	-	-
Primigravida	510	558	1.0		-	-
Mother’s parity						
Multipara	1,692	1,854	0.99 (0.83, 1.18)	0.934	-	-
Nullipara	522	564	1.0		-	-
Mother’s history of abortion						
Yes	144	138	1.07 (0.80, 1.44)	0.634	-	-
No	2,070	2,280	1.0		-	-
Mother’s history of stillbirth						
Yes	138	78	1.36 (1.05, 1.76)	0.020	-	-
No	2,076	2,340	1.0		-	-
Birth weight (g)	2214	2418	0.48 (0.42, 0.55)	0.001	0.39 (0.33, 0.46)	0.001
Type of road to the nearest health facility						
Asphalt	282	924	0.42 (0.32, 0.54)	0.001	-	-
Other	1,932	1,494	1.0		-	-
Walking distance to the nearest health post in minutes						
30+	1,344	732	1.90 (1.63, 2.22)	0.001	-	-
<30	870	1,686	1.0		-	-
Walking distance to the nearest health center in minutes						
40+	1,110	474	1.94 (1.68, 2.23)	0.001	-	-
<40	1,104	1,944	1.0		-	-
Walking distance to the nearest hospital in minutes						
60+	1,584	666	2.66 (2.23, 3.18)	0.001	2.66 (2.12, 3.35)	0.001
<60	630	1,752	1.0		1.0	

Significant at p<0.05; CI = confidence interval.

### Determinants of utilization of healthcare services

The Prentice, Williams, and Peterson’s total time Cox-type survival model analysis indicated that compared with younger mothers, older mothers with sick newborns (AHR = 1.22; 95% CI: 1.00, 1.50) or postpartum illnesses (AHR = 1.40; 95% CI: 1.03, 1.89) were more likely to seek healthcare.

## Discussion

Our study indicates that rural Ethiopian women and neonates experienced a high burden of postpartum and neonatal illnesses. For women, these illnesses included blurred vision with severe headache, excessive vaginal bleeding, severe abdominal pain, foul-smelling vaginal discharge, urinary incontinence, breast pain and engorgement, high fever, anemia, and hypertension. For neonates, these illnesses included feeding problems and diarrhea. All symptoms signal potentially dangerous illnesses in the community that must be properly diagnosed and treated. Yet, few received appropriate healthcare.

Our study involved symptom registration conducted by trained lay field workers. As such, translating these symptoms into diagnoses may be difficult. However, such symptoms can indicate a heavy illness burden in the community. For example, the estimated population of Gedeo in South Ethiopia is 1.1 million, and the crude birth rate is 31.8 of 1,000 live births [21]. Our study suggests that symptoms of potentially severe postpartum illnesses occur in about 31% of cases, and signs of severe neonatal illnesses occur in about 48% of cases. Accordingly, more than 10,800 women and 16,700 neonates in the Gedeo population should require postpartum and neonatal medical attention each year. Yet, even a smaller number of women and neonates requiring treatment would be a substantial burden on the already weak healthcare system [39]. It is therefore essential that efforts to address these issues ensure appropriate healthcare for ill women and neonates [40].

In this study, four in five women experienced at least one postpartum illness. This incidence rate was higher than in a previous study from rural India [41]. Many recurring episodes of symptoms were recorded during the study period, revealing a potentially high burden of postpartum illnesses in this area. This finding suggests that the healthcare system should be prepared for early identification and intervention.

The presence of many mildly anemic and a few severely anemic women might be due to excessive postpartum bleeding, lack of iron and folic acid supplementation, and pre-existing anemia.

Objectively measuring excessive vaginal bleeding is difficult in a survey setting, however, and clinical assessments tend to underestimate or overestimate actual volume loss. Nevertheless, one in five women has anemia, which can cause fatigue and other symptoms, and measuring hemoglobin levels is not commonly done in these communities. Similar to our previous study [22], we also demonstrated that most women did not receive iron or folate supplementation after childbirth, as advised by national policy. The burden of anemia in the community may serve as another indicator that the healthcare system should be strengthened.

The rate of postpartum illnesses in our study was lower than that found in studies from Pakistan [4] and India [6]. This gap could be due to differences in the study population, study design, and study setting. The studies from India and Pakistan included women either from home or healthcare facilities, whereas we did multiple visits at home and focused on both severe and minor forms of postpartum illnesses. However, the incidence of postpartum illnesses in our study was higher than that found in Jamaica, Kenya, and Malawi [7].

About 32% of poor women experienced one or more postpartum illnesses; however, fewer poor women received healthcare compared to rich women. The risk factors for postpartum illness were comparable to those from a previous study that documented women’s age at first marriage, women’s age at first birth, wealth index, and walking distance to the nearest healthcare institution as significant factors [8]. However, that study was based on cross-sectional data from healthcare facilities and did not focus on women from rural communities. Factors such as home delivery and parity also contribute to a high burden of postpartum illnesses [42]. Our study further suggests that age can influence the utilization of healthcare services [8]. This finding from our study needs further research to better reveal the factors related to illnesses after childbirth and to inform policy-makers.

In our study, the incidence of neonatal illnesses was higher than in recent studies from Bangladesh [15] and India [16]. This difference in incidence rates could be due to the study design, as our study was a cohort study, and the studies from Bangladesh and India were cross-sectional and assessed neonatal illnesses at a point in time. This might also be due to the high prevalence of home deliveries, in which many of the ill-neonates might not be followed, identified, treated, or referred to at the community level.

The common neonatal symptoms we reported include high fever, difficult or fast breathing, low temperature, poor sucking or feeding, chest in-drawing, and jaundice [16] which were in line with recent studies from Bangladesh [15] and India [16]. Factors such as geographic location and household wealth status [8] contribute to a high burden of neonatal illnesses. Our study further suggests that residence, low birth weight, type of road access, and walking distance were associated with neonatal illnesses.

The rate of low birth weight in this study is similar to other studies in Ethiopia [43] and was in our study expected to be a risk factor for neonatal illnesses. However, because few of the neonates with low birth weight sought healthcare, our study was underpowered to do such analyses.

The low rate of healthcare-seeking behavior remains a key challenge to improving neonatal health in developing countries [44]. In Bangladesh, less than 5% of neonates with illnesses visit a healthcare institution [45]. Contributing factors to this low rate include mother’s poor recognition of illnesses, distance to a healthcare facility, and financial constraints [46]. We similarly found that the mother’s perception of the severity of symptoms was another key challenge to seeking healthcare for her child, along with financial problems and beliefs that healthcare was not necessary, the latter of which may be due to previous exposure to poor or lacking healthcare services. Together, these findings suggest that efforts are needed to strengthen the provision of neonatal healthcare at the community level. Informing and teaching mothers and women about the use of health services is important. The health extension system combined with the local women’s associations and networks in their communities (kebeles) gives us an opportunity to improve the health-seeking habits of mothers.

Our study has some limitations. We included only those women who attended two or more antenatal care visits, which may have caused selection bias, as discussed in our previous study [22]. Thus, our study may not be fully representative, as women or neonates born to mothers who did not receive antenatal care could have a higher incidence of illnesses and lower utilization of healthcare services. Also, we did not register the type and quality of postpartum and neonatal healthcare services provided by the health extension workers.

Although women in Ethiopia usually schedule antenatal care in late pregnancy or not at all [21], we were able to include more than 75% of pregnant women in the study areas in our analysis. However, we investigated a limited range of disorders and did not collect information on vaginal tears, anal incontinence, voiding difficulties, hemorrhoids, sexual problems, wound breakdown, backache, and constipation. In general, our registration of symptoms was based on subjective reports by the women.

The main strength of the study is that the follow-up was done at the women’s homes or places they reside in. This study presents a prospective measurement of postpartum and neonatal illnesses in a rural area. This information has not been well documented at the community level and has been considered infeasible to study as it is considered as costly. Because the research teams consisted of residents of the same villages, they were well accepted by the population and could be present at the time of any illnesses.

Unlike previous studies, this study includes conditions during the pregnancy, postpartum, and neonatal periods. This coverage provides a comprehensive picture of health problems in women and neonates and helps inform policy decision-making. The target populations for these policies are similar to the study population concerning geographic, temporal, and ethnic variables. The findings of this study thus may generalize best to similar settings with poor and rich women and neonates, women and mothers of neonates with and without formal education, and women and mothers of neonates of different ages.

## Conclusions

The burden of postpartum and neonatal illnesses in rural communities in southern Ethiopia remains high. Unfortunately, a substantial proportion of ill neonates and women did not utilize healthcare services due to challenges in access to appropriate healthcare services. In terms of policy implications, our findings could serve as a basis for more detailed interventions in Ethiopia and provide useful insights concerning illnesses during the postpartum and neonatal periods. Low utilization of healthcare services appears to be an important area that needs immediate intervention.

Therefore, the efforts of the Ministry of Health should be directed towards strengthening the health system that enables in identifying, managing, treating, and referring maternal and neonatal illnesses and provide a reasonable level of healthcare at the community level. Interventions could include the promotion of health education that encourages women and mothers to seek appropriate and timely healthcare, increases the percentage of women and neonates receiving healthcare during illnesses, and ensures maternal and neonatal health during the postpartum and neonatal periods.

The Ethiopian health extension program was developed in a context in which maternal and neonatal health outcomes and coverage of essential healthcare services are very poor [18]. However, we did not study the healthcare system in the context of postpartum and neonatal illnesses. Thus, the content and quality of services provided by health extension workers at the community-level were not assessed. We also did not study the knowledge and skill of health extension workers in identifying and managing these illnesses and their related factors could constitute a potential future research area. For example, the tool used in our study could be used by health extension workers during their scheduled home visits to identify and assess illnesses among postpartum women and their neonates [33]. Therefore, more work is needed to enable the health system in identifying, managing, treating, and referring ill-women and neonates in need of healthcare services at the community level.

## Supporting information

S1 Fig The flowchart of the recruitment of participants. (TIFF)

S1 Table Symptoms of postpartum illnesses. (DOC)

S2 Table Symptoms of neonatal illnesses. (DOC)

S1 File Gedeo questionnaire for postpartum women and neonates. (RAR)

S2 File Amharic questionnaire for postpartum women. (RAR)

S3 File English questionnaire for postpartum women. (RAR)

S4 File Amharic questionnaire for neonates. (RAR)

S5 File English questionnaire for neonates. (RAR)
