# Supplementary figures and images for: Incidence of postpartum and neonatal illnesses and utilization of healthcare services in rural communities in southern Ethiopia: A prospective cohort study
Source: PLoS One. 2020 Aug 27;15(8):e0237852. doi: 10.1371/journal.pone.0237852 (PMC7451546; doi:10.1371/journal.pone.0237852)

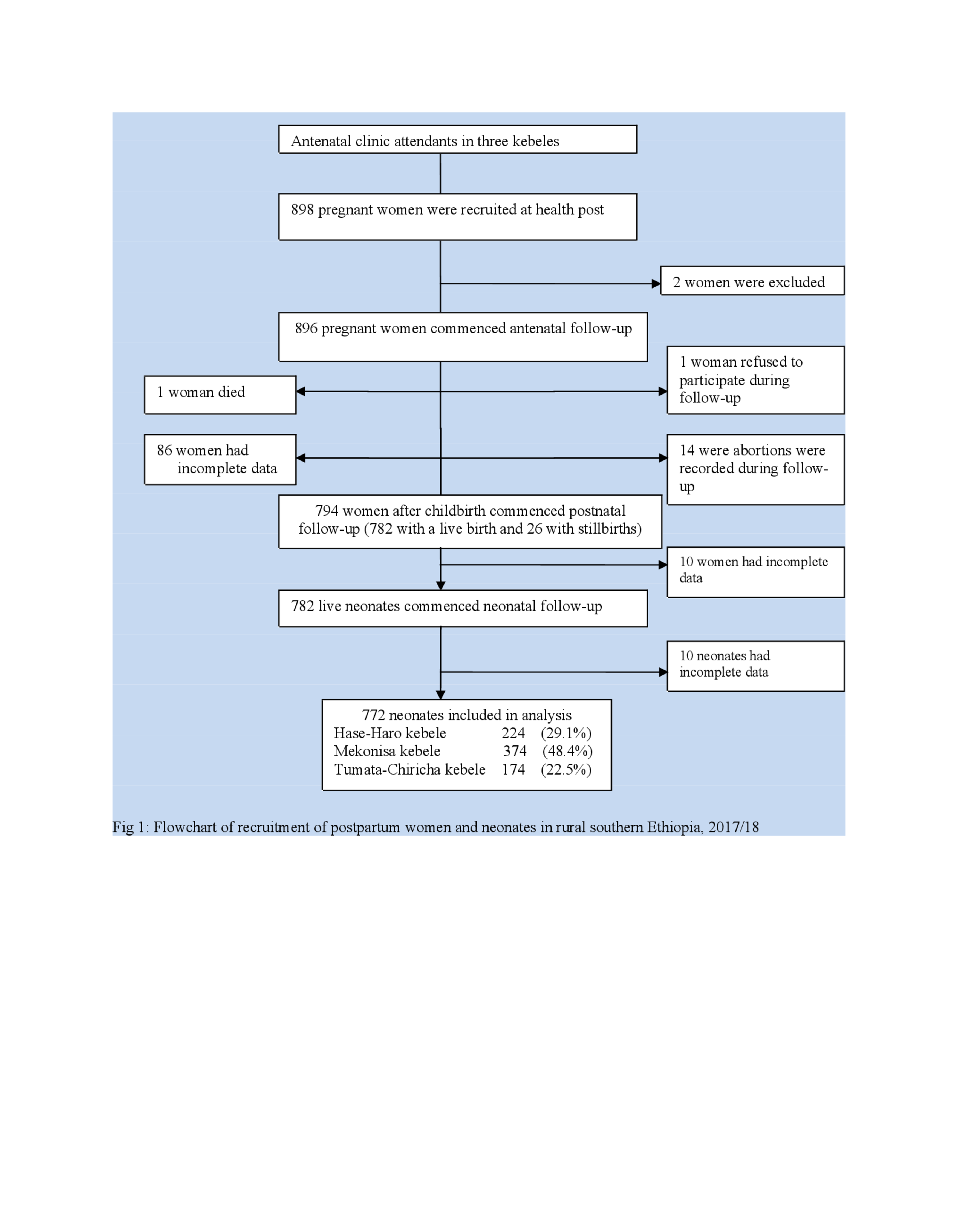

Supplement: S1 Fig — (TIFF) [file pone.0237852.s001.tiff]
