# Supplementary material for: Incidence of postpartum and neonatal illnesses and utilization of healthcare services in rural communities in southern Ethiopia: A prospective cohort study
Source: PLoS One. 2020 Aug 27;15(8):e0237852. doi: 10.1371/journal.pone.0237852 (PMC7451546; doi:10.1371/journal.pone.0237852)
Supplement: S1 Table — (DOC) [file pone.0237852.s002.doc]

**S1 Table: Sign and/or symptoms of postnatal illnesses**

| **Type** | **Symptoms** | **Signs** | **Definition** |
| --- | --- | --- | --- |
| Visual disturbances/blurred vision with headache | Hazy, blurred, or double vision. Seeing floating "spider webs" | Swollen, itching, burning, or discharge red eyes | A vision disorder with an impairment of the sense of vision |
| Excessive vaginal bleeding >2 or days | Contractions, abdominal pressure, cramping, and ache in the lower back | Spotting or discharge of blood from the vagina | A mother used > five pads a day or an increase in the use of pads (by at least 2). The blood has no difference in the colour from red to pink, then brown, and finally to a yellowish white. |
| Severe abdominal pain | Discomfort in the upper left or right; middle; or lower left or right abdomen | Swelling and tenderness when you touch the abdomen | Stomach ache |
| Foul vaginal discharge/itching : | Pain, itching, or burning around the vagina | The fluid that flows out the vaginal opening | A fluid with burning during micturition, rash, and/or odour due to infection of the genital tract |
| Urinary incontinence | **Pass urine unintentionally,** urge incontinence, and irritation of the perineal area | Anger, the decline in social activity, desire for attention, the odour of urine or faeces in the room, and soiling of undergarments or bed linens | Urine leaks when you exert pressure on your bladder by coughing, sneezing, laughing, exercising or lifting something heavy. |
| Painful, engorged breast or sore in breast | Breast problems after delivery or difficulties in breastfeeding | Engorged breasts can become extremely large, tight, lumpy, and tender.  The skin appearing shiny and diffusely red. The nipples may become stretched tight and flat | Breast engorgement is the development of hard, swollen, painful breasts from too much breast milk.  Either cracked nipples or painful lumps in breast or difficulties in breastfeeding. |
| Fatigue/Tiredness | Headache, dizziness, aching muscles | Sleepiness, slowed reflexes and responses, impaired decision-making and judgment | A subjective feeling of tiredness |
| High fever | Headache | Sweating, chills and shivering | Temperature > 37.5 °C on any day |
| Anaemia | Easy fatigue and loss of energy, shortness of breath and headache, difficulty in concentrating dizziness | Unusually rapid heart beat, pale skin. | A condition that develops when your blood lacks enough healthy red blood cells or haemoglobin and its measurement lower than an accepted normal value |
| Hypertension | Severe headache, fatigue or confusion, vision problems, pounding in chest, neck, or ears. | Difficulty breathing. Irregular heartbeat | Either a systolic or a diastolic blood pressure measurement higher than an accepted normal value |
