# Supplementary material for: Incidence of postpartum and neonatal illnesses and utilization of healthcare services in rural communities in southern Ethiopia: A prospective cohort study
Source: PLoS One. 2020 Aug 27;15(8):e0237852. doi: 10.1371/journal.pone.0237852 (PMC7451546; doi:10.1371/journal.pone.0237852)
Supplement: S2 Table — (DOC) [file pone.0237852.s003.doc]

**S1 Table:** Sign and/or symptoms of neonatal illnesses

| **Type** | **Symptoms** | **Signs** | **Definition** |
| --- | --- | --- | --- |
| **Diseases during the neonatal period** | | | |
| Not sucking properly | The baby cries excessively as he/she is hungry, gaining weight too slowly or not gaining at all, and may sleep on the breast, but cry if taken away from the breast | The baby’s low output of urine and stools | Baby breastfeeding problems |
| Fast breathing | Grunting, coughing | Rapid or irregular breathing, flaring nostrils, retracting | Fast breathing rate ≥60 per minute |
| Difficult to wake up | Unexplained decrease in daytime performance, breathing pauses during sleep | Difficulty to wake for a feeding | Difficult to wake up as the bBaby is unusually quiet or baby is sluggish when awake |
| No spontaneous movement | Not sucking, crying, and swallowing | No spontaneous reflexes | The baby has no spontaneous reflexes such as sucking, crying, and swallowing |
| Infected cord | Pain, pus from the umbilical stump, fever | Redness, warmth, swelling, fast heart rate | The cord is infected or with pus within the first two weeks |
| Severe chest in-drawings | A sign of respiratory distress | The inward movement of the lower chest wall (i.e., ribs) when the child breathes in | The inward movement of the lower chest wall (i.e., ribs) when the child breathes in and is a sign of respiratory distress |
| High fever | Headache | Sweating, chills and shivering | Temperature > 37.5 °C on any day |
| Low body temperature | Slurred speech or mumbling, drowsiness or very low energy, confusion or memory loss, loss of consciousness | Shivering, slow, shallow breathing, Weak pulse, bright red, cold skin | Temperature < 35.5 °C on any day |
| Eye infection | Pain or discomfort, itchy eyes, feeling that something's on or in your eye, light sensitivity, burning in your eyes | The eyelid is tender when you touch it | Red eye or discharging eye |
| Jaundice | fatigue, dark urine, joint and muscle pain, loss of appetite, fever, abdominal discomfort, weakness and | Yellowish whites part of the eyes (sclera) and skin | Yellowish eye, skin, palms and soles |
| Lethargy | To have little or no energy, feel sleepy or fatigued and sluggish | Sleepy or fatigued | To have little or no energy, are drowsy or sluggish, and may sleep longer than usual |
| Diarrhoea | Frequent loose, watery stools.  Abdominal cramps. Abdominal pain. | Loose, watery stools | Having 2 or more watery or very loose stools |
| Persistent vomiting | Feel nauseous, and abdominal pain | Gagging, retching, choking, involuntary stomach reflexes, the mouth filling with saliva, and need to move or bend over | Forceful throwing up of stomach contents through the mouth |
